# Characterisation of Hemp Fibres Reinforced Composites Using Thermoplastic Polymers as Matrices

**DOI:** 10.3390/polym14030481

**Published:** 2022-01-25

**Authors:** Lucia Stelea, Ioan Filip, Gabriela Lisa, Mariana Ichim, Mioara Drobotă, Costică Sava, Augustin Mureșan

**Affiliations:** 1 Faculty of Industrial Design and Business Management, Gheorghe Asachi Technical University of Iasi, D. Mangeron Street, No. 29, 700050 Iasi, Romania; luci.stelea@taparo.ro (L.S.); mariana.ichim@academic.tuiasi.ro (M.I.); costica_sava@yahoo.com (C.S.); 2 Taparo Company S.A.,198 Borcut Street, 435600 Targu Lapus, Romania; ioan.filip@taparo.ro; 3 Faculty of Chemical Engineering and Environmental Protection, Gheorghe Asachi Technical University of Iasi, 700050 Iasi, Romania; gapreot@ch.tuiasi.ro; 4 Petru Poni Institute of Macromolecular Chemistry, 41A Grigore Ghica Voda Street, 700487 Iasi, Romania; miamiara@icmpp.ro

**Keywords:** composites, hemp reinforcement, polymer matrix, thermal stability, FTIR, SEM, colour strength

## Abstract

Hemp fibres used as a reinforcing agent and three polymeric matrices (polypropylene, bicomponent, recycled polyester) were used to obtain composite materials by needle punching and heat pressing. The influence of the hemp/matrix ratio and the nature of the matrix on the properties of the composites were analysed. The obtained composites were characterised by physical–mechanical indices, thermal analysis (thermogravimetry (TG), differential thermogravimetry (DTG) and Differential Scanning Calorimetry (DSC)), Fourier Transform Infrared Spectroscopy (FTIR-ATR) analysis, Scanning Electron Microscopy (SEM) and Chromatic measurements. The mechanical properties of composites are influenced by both the hemp/matrix ratio and the nature of the matrix. The thermal stability of composites decreased as the amount of hemp increased (for the same mass losses, the decomposition temperature decreased significantly for composites containing a quantity of hemp greater than 50%). Regarding the nature of the matrix, for the same mass loss, the highest decomposition temperature was presented by the composites containing recycled polyester as matrix, and the lowest one was presented by composites containing polypropylene fibres as matrix. The FTIR and SEM analyses highlight the changes that occurred in the structure of the composite, changes determined both by the amount of hemp in the composite and by the nature of the matrix.

## 1. Introduction

In recent decades, due to their wide range of properties, composite materials have successfully replaced traditional materials such as metals, wood and plastics in many industrial fields: aerospace, automotive, energy, construction, marine, transportation, infrastructure, electronics and electrotechnics, agriculture, food, chemical, furniture, packaging, sports and recreation [1,2,3,4,5,6,7]. The rapid growth of the composite material market is closely related with performance limitations of traditional materials, such as the availability and the price. Composite materials combine the qualities of two or more materials with different properties in a product that has superior characteristics over the individual constituents. One constituent is called matrix, and embeds the other constituent, called the reinforcement. The reinforcement takes over the load and gives the composite material strength and rigidity, and the matrix binds the reinforcements together and transfers the load to and between the reinforcements. Polymer composites reinforced with natural lignocellulosic fibres are intensively studied as replacements in common applications for composites reinforced with synthetic fibres (glass, carbon, aramid) because of the environmental and economic disadvantages that the latter have [8,9,10]. Synthetic fibres are costly and non-biodegradable, their production is energy consuming, and the composites obtained from them are not easily recycled. In comparison with synthetic fibres, natural fibres are cheap, biodegradable, renewable, recyclable, nontoxic, and easily available. They have low density, good mechanical properties and do not degrade the processing equipment [11,12,13,14].

At present, composite materials obtained from polymeric matrices reinforced with natural cellulose fibres are particularly important due to their ecological nature, biodegradability, low price and high mechanical properties. These materials must meet several basic conditions: they must be completely combustible and biodegradable. To compensate for some deficiencies of natural fibres, such as their lower thermal stability, increased water absorption capacity and less incompatibility with the polymer matrix, researchers are constantly discovering new methods for obtaining composite materials with new properties. In the future, these composite materials are expected to play an increasingly important role [15].

Among natural fibres, hemp is one of the strongest and stiffest, fibreand therefore it has an enormous potential to be used as a reinforcement in composite materials [16].

In addition, industrial hemp (*Cannabis sativa*) seems to be one of the most profitable crops. One hectare of hemp is the equivalent of four hectares of forest, when we talk about the pulp used to make paper. The vegetation period of hemp is about 100 days, which does not compare to the time required to plant and obtain cellulose from the trees of a forest.

Hemp fibre is a plant that during growth produces an amount of oxygen equal to the amount of CO_2_ that is released when burning the same amount of hemp.

In Europe, in recent years, hemp production has increased considerably from 94,120 tons in 2015 to 152,820 tons in 2019, an increase of 62.4% [17,18]. Due to its positive impact on the environment and high yield, hemp is a valuable crop for producers. Taking into account that the farmers from European Union countries may benefit from support for hemp cultivation, one can say that hemp production has shown an upward trend, which makes it easy to use and available for industrial applications. Environmental concerns and hemp’s unique characteristics have boosted research concerning the use of hemp fibres as reinforcement in composites. Hemp fibres can be used successfully to obtain biocomposites [19,20].

It can be stated that hemp fibres can be used in obtaining composite materials for multiple uses. By using various treatments, the of a matrix–hemp fibre interface can be improved. By properly choosing polymeric matrices, composite materials can be obtained that can be totally or partially biodegradable, and sometimes with a level of low moisture absorption [21,22].

Studies regarding the properties and applications of composite materials obtained using hemp fibres as reinforcing agents and various matrices, such as polyethylene [23,24,25], cement [26,27], polyurethane [28], polylactic acid [29] and polypropylene [30,31,32,33,34,35,36], are presented in the literature. Different authors have highlighted compression molding, extrusion, and injection molding as technologies suitable for processing the composites [37,38,39,40,41,42].

The processing of hemp fibres is influenced by the processing temperature, and the upper limit is conditioned by the temperature at which the degradation of cellulose and its companions begins [43,44,45,46].

The aim of this paper was to obtain and characterise composite materials consisting of hemp fibres and three different polymer fibres used as matrices: polypropylene (PP), bicomponent fibres (BI) and recycled polyester fibres (PES), which have melting temperatures lower than the degradation temperature of hemp. The composite materials were obtained using various mixing ratios of hemp fibres with the three polymer matrices. The resulting materials were characterised by their mechanical properties, thermogravimetric analysis (TG/DTG), Differential Scanning Calorimetry (DSC), Fourier Transform Infrared Spectroscopy (FTIR), Scanning Electron Microscopy (SEM) and chromatic measurements.

## 2. Materials and Methods

### 2.1. Materials

Hemp fibres with a reinforcing role and three chemical fibres with a matrix role were used for the study. The virgin PP used in this work was purchased from EurocomfilsrlTg. Lapus, Romania, being produced by the Beauileu International Group, Kruisem, Belgium. It has the following characteristics: melting flow index (MFI) = 7.5 g/10 min (230 °C/2.16 kg); density = 900 kg/m^3^; melting temperature = 160 °C. The bicomponent fibre (produced by the company Far Eastern Textile LTD, Taiwan) is of type C/C (cover/core) with a polypropylene sheath and polyester (poly(ethylene terephthalate) core purchased by SC Taparo SA, Tg. Lapus, Romania. The recycled polyester fibres (poly (ethylene terephthalate) were produced by Green Group Fibre Company, Buzau, Romania by recycling polyester bottles. The hemp fibres were purchased from SRL Pianu de Jos Company, Alba County, Romania. The lengths of the fibres in the bales range between 5 and 25 cm. The fibres were cut to 5–6 cm lengths in order to be used.

The main characteristics of the fibres used in composites are presented in Table 1.

### 2.2. Experimental Variants

In order to obtain composite materials, hemp fibres (H) as reinforcing agent and three types of thermoplastic polymer fibres as matrix were used: polypropylene (PP), bicomponent fibre (BI) and recycled polyester fibres (PES). Within each variant the percentage composition of the two components was modified from 0 to 100%. The coding and composition of the materials according to the experimental variants are presented in Table 2.

### 2.3. Composites Processing

The manufacturing process of composite materials consisted of the following steps:

- Manufacturing of needle-punched nonwoven fabrics;
- Overlapping of nonwoven fabrics. Four samples of each nonwoven fabric variant have been overlaid alternatively in the longitudinal and transversal directions;
- Thermoforming. The overlaid nonwovens were placed in the mould of the thermoforming machine and then heated and pressed between the plates until the polymer matrix melted. The material was cooled in the rectangular mould (temperature—190–240 °C, pressure—735.46 MPa, pressing time—15 min and cooling time—15 min).

### 2.4. Characterisation

#### 2.4.1. Mechanical Properties

In accordance with the specifications of EN 326-1 standard, type 2 samples (250 mm length, 25 mm width) were used for tensile testing. The distance between clamps was 150 mm and the testing speed was 2 mm/min as specified in SR EN ISO 527-4:2000 standard. The tensile test was performed on a ZWICK tensile tester (Ulm, Germany) at 23 °C according to ISO 527-4 standard. Five repetitions were done for each sample.

#### 2.4.2. Thermogravimetric Analysis (TG/DTG)

The thermogravimetric measurements were performed using a Mettler Toledo TGA/SDTA 851 balance (Columbus, OH, US), allowing the simultaneous recording of the weight losses (TG) and the derivative thermogravimetric curves (DTG). The analysis was carried out under constant nitrogen flow (20 mL/min), at a heating rate of 10 °C/min. The heating scans were performed on 1.9–5.3 mg of sample in the temperature range 25–700 °C.

#### 2.4.3. Differential Scanning Calorimetry (DSC)

The melting and crystallisation behaviour of the composites was studied using a Mettler Toledo DSC1 differential scanning calorimeter (Columbus, OH, US). The mass of samples encapsulated in aluminium pans with pierced lids to allow evaporation of the volatile components ranged between 2.2 and 5.4 mg. A nitrogen flow rate of 150mL/min was used. The sample was initially scanned from −60 to 200 °C or −60 to 300 °C at a heating rate of 10 K/min and followed by cooling with the same rate, and then it was rescanned in the same temperature interval. The crystallisation temperature (Tc), the crystallisation enthalpy ($H_{c}$), the melting temperature (Tm) and the melting enthalpy ($H_{m}$) were obtained from the heating–cooling–heating cycle of the sample. The crystallinity, ${Χ}_{c}\left(\%\right)$, of the different compounds was obtained by Equation (1).

(1) $${Χ}_{c}\left(\%\right)=\left[\frac{\Delta H_{m}}{\Delta H_{m}^{0}\cdot \left(1-w\right)}\right]\cdot 100$$

where in $\Delta H_{m}^{0}$ corresponds to the theoretical melting enthalpy of the fully crystalline polypropylene, whose value according to the literature’s data is 207 J∙g^−^¹, and w is the mass fraction of the filler [47].

#### 2.4.4. Fourier Transform Infrared Spectroscopy

For the spectroscopic studies, a Bruker FTIR Vertex 70 spectrometer equipped with a diamond ATR (attenuated total reflection) device (Golden Gate; Bruker, Billerica, MA, USA) and ATR-FTIR (attenuated total reflection Fourier transform infrared) was used. The ATR-FTIR spectra were measured in the spectral range of 800–4000 cm^−1^, by the accumulation of 64 scans.

#### 2.4.5. Scanning Electron Microscopy

The scanning electron microscopy studies were performed on samples fixed on aluminium supports. The surface morphology of the uncoated samples was examined using an SEM-type Quanta 200 (FEI) (Hillsboro, OR, USA), operating at 20 kV with secondary electrons in the low vacuum mode (large-field detector (LFD)), the magnification being indicated on the micrographs.

#### 2.4.6. Chromatic Measurements

Samples of composite materials were subjected to colour measurements performed on the entire spectrum of the visible range. The sample were evaluated for colour strength in terms of Kubelca–Munk equation [48]

(2) $$\frac{K}{S}=\frac{{\left(1-R\right)}^{2}}{2R}$$

wherein “R” is the reflectance at sample complete opacity;

“K” is the absorption coefficient;

“S” is the scattering coefficient.

A DATACOLOR SF-300 spectrophotometer (Lawrence Township, NJ, USA) was used for this purpose. The processing of the experimental data was performed with the help of some specialised software, Micromach^R^2000.

## 3. Results and Discussions

### 3.1. Mechanical Properties

**Figure 1:**
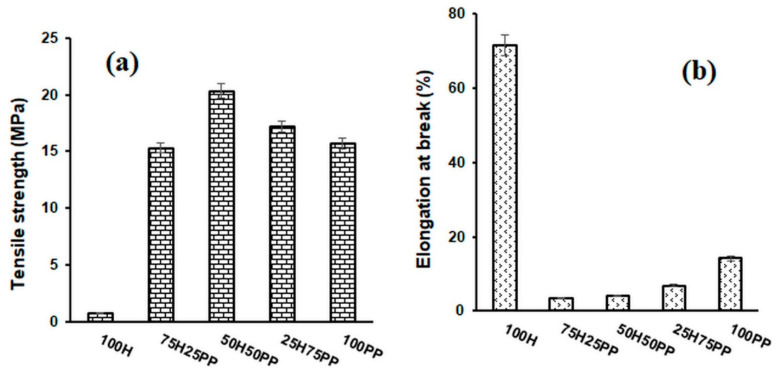
Variation in tensile strength (**a**) and elongation at break (**b**) for hemp and polypropylene–fibre composites.

**Figure 2:**
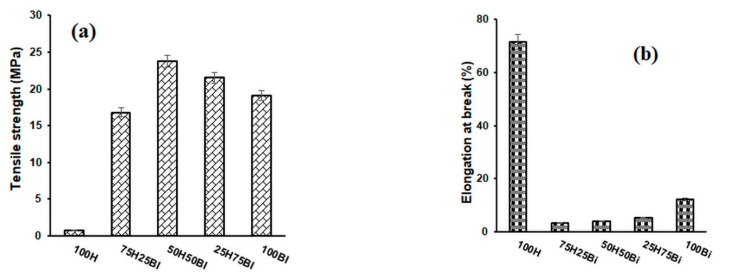
Variation in tensile strength (**a**) and elongation at break (**b**) for hemp composites and bicomponent fibres.

**Figure 3:**
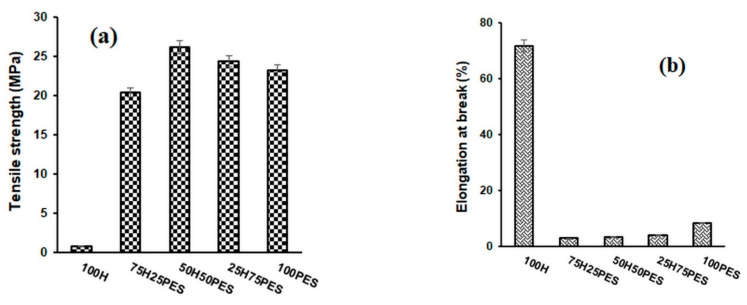
Variation in tensile strength (**a**) and elongation at break (**b**) for composite materials of hemp and polyester fibres.

The mechanical properties of the fibres used as reinforcing agents, of the polymeric fibres used as matrices, and of the composite materials obtained from them are shown in Figure 1, Figure 2 and Figure 3. The 100% hemp sample, which is a nonwoven material, has a much lower tensile strength (0.76 MPa) than composite materials. The tensile strengths of composite materials containing hemp fibre as a reinforcer increased as the content of the polymer matrix increased. The highest values were obtained for the composites that contain a quantity of hemp ranging between 30 and 50% (*w*/*w*), after which there was a slight decreasing tendency of the tensile strength. Regarding the nature of the matrix, the highest values of tensile strength were obtained for composite materials with polyester matrix (26.2 MPa), followed by composite materials with bicomponent fibre (23.77 MPa) and then by composite materials with polypropylene (20.33 MPa).

The elongation at break of the 100% hemp nonwoven material was approximately 71.5%, and this decreased in the composite materials as the hemp content increased. This decrease can be explained by the increased crystallinity of these composite materials (Table 4). The elongations at break of the 100% polymer matrices are higher than those of the composite materials. PP has the highest value of elongation at break (14.1%), being followed by BI (12.5%) and afterthen by PES (8.3%). These values confirm the role of the reinforcing agent used in obtaining the composite materials.

### 3.2. TGA and DTG Analysis

**Figure 4:**
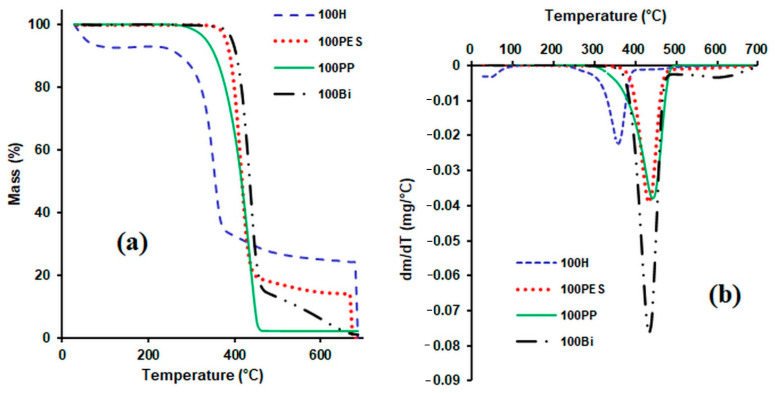
TG curves (**a**) and DTG curves (**b**) for hemp fibres and matrix polymer fibres.

**Figure 5:**
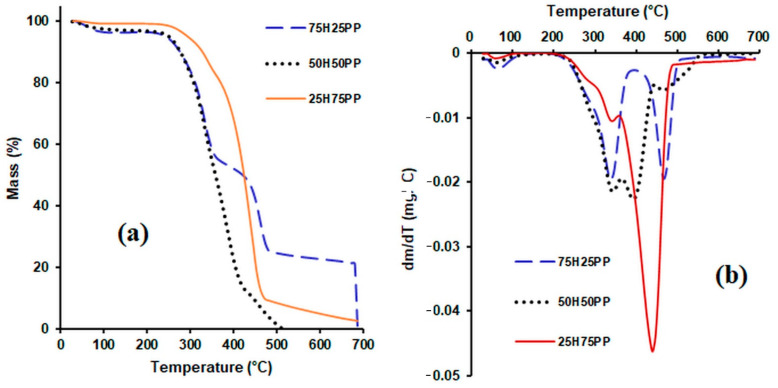
TG curves (**a**) and DTG curves (**b**) for composite materials consisting of hemp fibres and polypropylene fibres.

**Figure 6:**
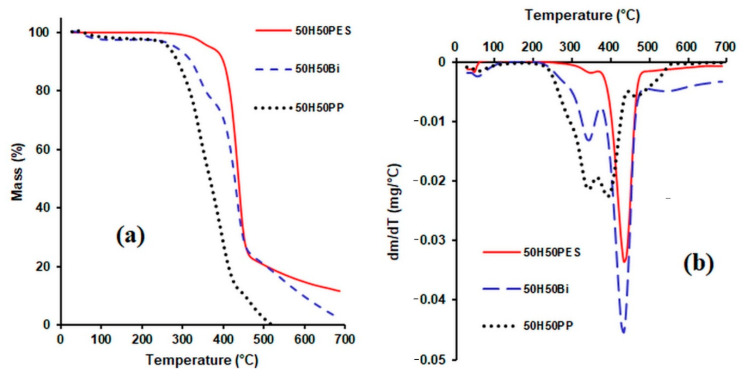
TG curves (**a**) and DTG curves (**b**) for composite materials consisting of hemp fibres and various matrices.

The TGA and DTG thermograms of the hemp fibres used as reinforcers, of the various matrices (formed of polypropylene fibres, polyester fibres and bicomponent fibres) and of the composites obtained from them are presented in Figure 4, Figure 5 and Figure 6.

Before analysing the thermal behaviour of composite materials, it is important to analyse the thermal behaviour of each component of the composite (Figure 4). When analysing the TG curve of hemp fibre, a mass loss of 7.3% was observed in the temperature range between 41 and 149 °C, which can be attributed to the loss of moisture from the surface of hemp fibre. In the range 149–303 °C, the mass losses were small (7.06%). The highest mass loss of 57.7% occurred within the temperature range of 303 °C to 376 °C, and can be attributed to the degradation of cellulose and hemicellulose (the rate of degradation is greatest at 359 °C). At temperatures between 376°C and 498°C and even higher, there was a new mass loss due to lignin degradation [49].

Regarding the DTG curve, the peak at 55 °C can be attributed to the moisture loss of the hemp fibre, while the sharp peak at 559 °C is due to the degradation of cellulose and hemicellulose. The peak at 446 °C can be attributed to lignin degradation. [47].

From the TG diagram of the polyester fibre, it is observed that it did not undergo significant changes up to the temperature of 399 °C. In the temperature range 399–461 °C, there was a significant degradation of the fibre (mass loss of 89.33%). Degradation continued up to temperature of 650 °C. From the DTG curve, it can be observed that the degradation rate reached its maximum at 434 °C.

By analysing the thermal behaviour of the bicomponent fibre, which is made of PP (cover) and polyester (core), it was observed that up to the temperature of 396 °C, there were no significant changes in terms of weight loss. A significant degradation of the fibre occurred in the range 396–462 °C (mass loss of 84.97%). The degradation continued in the range 462–664 °C, with a mass loss of 14.81%.

The DTG curve of the bicomponent fibre indicates a maximum degradation at the temperature of 436 °C, which can be attributed to the degradation of the polypropylene component, and another one at 601 °C, probably due to the degradation of the polyester component.

From the TG diagram of the polypropylene fibre, it can be observed that a maximum degradation of the fibre occurred in the range 358–469 °C, with a mass loss of 98.14%. From the DTG curve, it can be observed that the maximum degradation of fibre took place at the temperature of 442 °C.

After analysing the TG curves of composite materials containing hemp as the reinforcing agent and polypropylene as the matrix (Figure 5), it can be observed that: in the first stage of degradation (between 25 and 150 °C), the mass loss increased with the increase in the hemp content, as would be expected, since the polypropylene matrix has a strong hydrophobic character and does not absorb moisture.

The thermal stability of the analysed composite materials decreased slightly with the increase in the hemp content used as reinforcing agent.

From the TG curves (Figure 6) obtained for the composite materials containing 50% hemp and 50% matrix (PP, BI and PES), it can be noticed that the thermal stability of the samples decreased in the following order: composites with polyester fibre matrix > composites with bicomponent fibre matrix > composites with polypropylene matrix. For temperatures higher than 420 °C, the composites with polyester fibre matrix and the composites with bicomponent fibre matrix showed an almost similar behaviour. From the analysis of the DTG curves it can be observed that four peaks appear in the composite material with the PP matrix. The first peak that appears in the range 54–120 °C corresponds to the loss of moisture, while the peak at 342 °C corresponds to the degradation of hemp, the peak at 393 °C can be attributed to the degradation of lignin and PP, and the one at 465 °C corresponds to the degradation of PP. The temperature range indicated for the use of this composite is 20–120 °C.

For the samples with bicomponent fibre matrix, three peaks occurred. The peak in the range 48–95 °C can be attributed to the moisture loss of the hemp fibre. The peaks from 344 °C and 432 °C can be attributed to the degradation of the hemp fibre and to the polypropylene component of the bicomponent fibre.

Three peaks appear in the DTG diagram of the composite with a polyester matrix. The peak in the range 54–74 °C can be attributed to the moisture loss from the hemp fibre, the one at 344 °C corresponds to the degradation of the hemp fibre, and the peak at 435 °C can be attributed to the degradation of the polyester fibre in the composite material.

The most important thermogravimetric characteristics (T_onset_—the starting temperature of the step, T_peak_—the temperature corresponding to the maximum value of the mass loss, T_endset_—the ending temperature of each step and w%—the percentage mass loss corresponding to each interval) are listed in Table 3.

Thermal stability increases as the amount of hemp in the composite decreases. For the same mass losses, the decomposition temperature decreased significantly for the composites that contain hemp in quantities higher than 50% (Table 4).

Regarding the influence of the matrix on the thermal stability of the composite, it can be observed that the composite material with the polyester fibre matrix exhibited the highest thermal stability, being followed by the composite with a bicomponent fibre matrix. For the same mass losses, the decomposition temperatures of the composites decreased in the order of 50H50PES > 50H50BI > 50H50PP.

### 3.3. DSC Analysis

**Figure 7:**
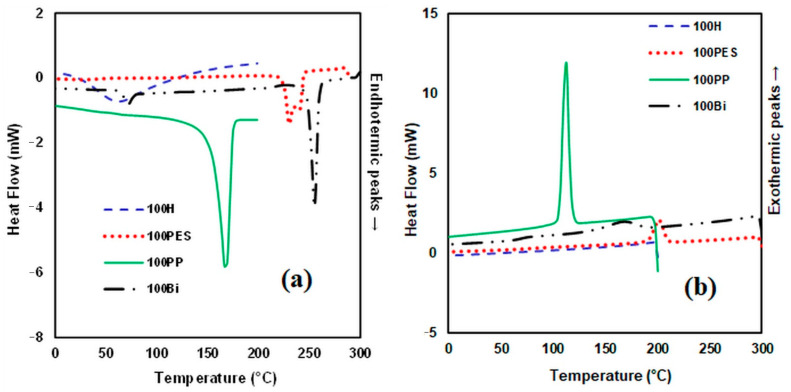
DSC curves heating process (**a**) and DSC curves cooling process (**b**) for hemp fibres and various matrices.

**Figure 8:**
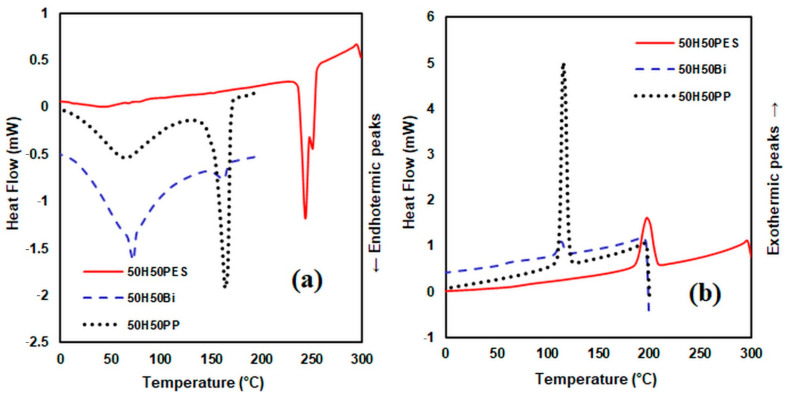
DSC curves heating process (**a**) and DSC curves cooling process (**b**) for composite materials consisting of hemp fibres and various matrices.

**Figure 9:**
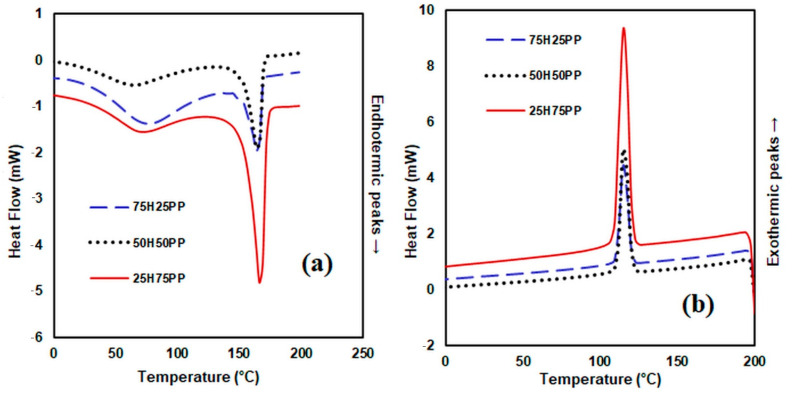
DSC curves heating process (**a**) and DSC curves cooling process (**b**) for the composite materials consisting of hemp fibres and polypropylene matrix.

The DSC thermograms indicate the melting and crystallisation temperatures of the analysed samples (Figure 7, Figure 8 and Figure 9). From the DSC curves (Figure 7a) corresponding to the heating process, it can be observed that the DSC curve of the hemp fibre shows a low endothermic peak around 63 °C, which corresponds to the decrease in moisture in the fibre. The DSC curve of polypropylene fibre presents an endothermic peak at 165 °C, while the DSC thermogram of the bicomponent fibre shows two peaks, the first one at 71 °C corresponding to the fibre in the mantle structure, and the second one at about 237 °C corresponding to the fibre core. In the case of the polyester fibre, the initial melting temperature is 235 °C, with a maximum of 239 °C and a final temperature of 243 °C. The DSC curves obtained in the cooling process (Figure 7b) indicate for hemp fibres an exothermic heat flow that is almost constant in the analysed temperature range. For the other fibres subjected to the cooling process, the crystallisation process takes place at 112 °C for polypropylene, at 166 °C for the bicomponent fibre and at 201 °C for the polyester fibre, respectively.

Regarding the nature of the matrix derived from the analysed composites (Figure 8), from the DSC curves, it can be observed that the first peak appears at 62 °C for the composite 50H50BI, at 72 °C for the composite 50H50PP and at 78 °C for the composite 50H50PES. These peaks are assigned to the endothermic processes that correspond to the evaporation of moisture from the composite material. The second peak occurs at 159 °C for 50H50BI, at 166 °C for PP and 248 °C for polyester fibre, respectively. From the diagrams resulting from the cooling process, the exothermic crystallisation peaks appear at 115 °C for 50H50PP, at 113 °C for 50H50BI and at 197 °C for 50H50PES, respectively. The composite materials obtained from 25H75 PP and 50H50PP have a higher melting temperature compared to the melting temperature of PP, while composites obtained from 75H25 PP have a lower melting temperature compared to the melting temperature of PP fibres. This could be explained by an increased interaction between the PP matrix and the reinforcer for composites containing up to 50% H. These results are also correlated with the results obtained in the case of physical–mechanical indices when the tensile strength increases up to a composition of about 50% hemp fibres, after which it shows a decreasing tendency.

Regarding the influence of hemp fibre content (Figure 9a) in polypropylene fibre matrix composites, the DSC curves are consistent with the TGA curves. The first endothermic peak appears at 70 °C for composite 25H75PP, at 72 °C for composite 50H50PP and at 75 °C for composite 75H 25PP, respectively.

From the diagram of the cooling process of the composite materials, we can observe an increase with a 2–3 °C increment in crystallisation temperature (Tc) of the composites compared to pure PP.

The crystallisation temperature of PP (112.5 °C read from the cooling curve of Figure 9b) suggests that PP is a semicrystalline polymer that exhibits a high tendency to crystalise.

The values of the crystallisation temperatures corresponding to the obtained composite materials are close. The difference between the crystallisation temperature of PP and the crystallisation temperatures of the obtained composites is around 3 °C, while the values of their crystallisation temperatures range between 112.5 and 115.55 °C.

This fact confirms that the hemp fibres acted as nucleation agents, and consequently the PP from the composite materials started to crystallise at a temperature higher than 112.5 °C, the surface of the hemp fibres constituting crystallisation centers for the polymeric matrix [50].

From Figure 7a and Figure 9a and from Table 5, it can be noticed that during the heating process, a slight decrease in the melting temperature of the composite material, Tm, occurs, compared to the melting temperature of the polymeric matrix. This behaviour was previously reported by other researchers [51,52].

This decrease in the melting temperature can be explained by the incompatibility between the PP fibre, which has nepolar groups, and the hemp fibre, a hydrophillic ligno-cellulosic fibre (between the two types of fibres only weak interactions occurred).

A second scan for the composite samples was also performed (with a second heating). The results obtained from the interpretation of the curves recorded at the second heating, the glass transition temperatures included (T_g_), have been inserted in the tables (Table 5 and Table 6).

For the composite materials obtained from PP and hemp, we can observe, on the one hand, an increase in the enthalpy of melting ΔH_m,_ and on the other hand a decrease in the crystallinity as the hemp fibre content from the composite decreased. Regarding the enthalpy of crystallisation, the higher the degree of crystallinity of the composite material, the higher the amount of heat released [53,54].

Table 5 shows the values obtained for the crystallisation enthalpy **ΔH_c_**, the melting enthalpy **ΔH_m_** and the level of crystallinity Χc, calculated with Equation (1). The value obtained for the melting enthalpy ΔH_m_ of PP is 74.4 J∙g^−1^, and close values were previously reported by other authors (∆Hm 74 (J∙g^−1^) [55]. It can be noticed that the value of crystallinity Χc for PP (45.19%) is lower compared to the values of the crystallinity level for the composite materials, which vary from 77.23% to 46.58%. From the curve recorded at the second heating, it is clear that the melting temperature **T_m_** of pure PP was 164.74 °C, while the characteristic melting temperatures of the composite materials ranged from 161.58 °C to 163.20 °C. The values of the crystallinity level Χc corresponding to the heating process ranged between 40.85% and 68.94%. Both for the cooling process and the heating process, the values of the crystallinity level for the composite materials were higher than the crystallinity level of pure PP used as matrix. These results could be explained by the nucleation effect exerted by the lignocellulosic hemp fibres on the PP matrix, during the manufacturing of the composite materials. The obtained results regarding the influence of the matrix type features on the composite materials containing 50% hemp and 50% polymer fibre are shown in Table 6.

The values for the melting enthalpy ∆Hm are higher for the polyester fibre than for the bicomponent fibre. Additionally, the values of the melting enthalpy ∆Hm of the composite materials obtained from these fibres are lower than those obtained for the polymer matrices.

The values of the glass transition temperature for recycled PES previously reported by other researchers range between 60 and 80 °C [56,57,58,59]. The Tg value obtained by us for recycled PES falls within this range.

### 3.4. FTIR Analysis

**Figure 10:**
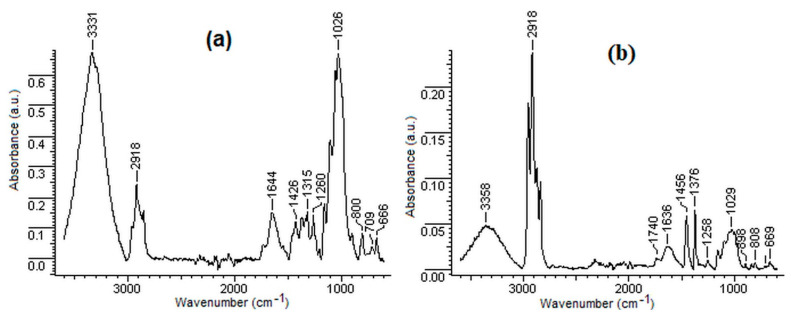

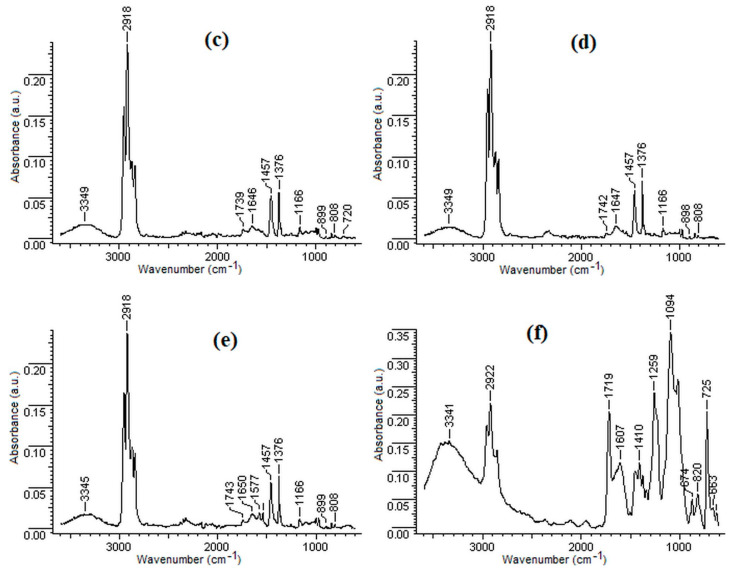

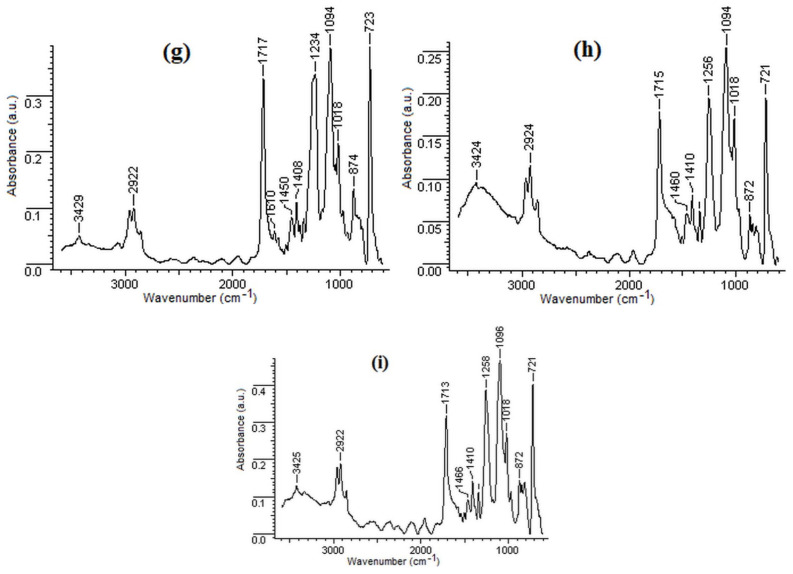
FTIR spectra of (**a**) 100H; (**b**) 75H25PP; (**c**) 50H50PP; (**d**) 25H75PP; (**e**) 100PP; (**f**) 50H50BI; (**g**) 100BI; (**h**) 50H50PES and (**i**) 100PES.

The FTIR spectra of the hemp and polypropylene, and of the composite materials for the wave numbers range (3600–600 cm^−1^), are shown in Figure 10a–e.

The broadband in the range 3429–3349 cm^−1^ is assigned to the stretching vibrations of the –OH groups belonging to the cellulose and hemicellulose from the hemp fibre [60].

The peaks located within the range 2963–2856 cm^−1^ in the FTIR spectra of the hemp, the polymeric fibres and the composite materials can be assigned to both types of stretching vibrations (symmetrical and asymmetrical) of the C–H bonds (CH_2_ groups) from the hemp and from the polymeric fibres [61].

The absorption band located in the range 1733–1741 cm^−1^ corresponds to the stretching vibrations of the C=O bonds presented in the lignin, pectin and waxes contained by the hemp fibre. The intensity of this absorption band decreased with the decrease in the hemp content in the composite materials wherein polypropylene was used as the matrix. In the case of polyester fibres, of the bicomponent fibres and of the composite materials containing these fibres as matrices, the peaks located at 1713 cm^−1^, 1717 cm^−1^, 1715 cm^−1^ and 1719 cm^−1^ are due to stretching vibrations of the group C=O from the polyester.

The intensity of the absorption band around 1640 cm^−1^, attributed to the bending vibrations of O–H bonds from the adsorbed water molecules, decreased once the amount of polypropylene from the composites had increased. This is due to the fact that by decreasing the amount of hemp there are fewer “free” hydroxyl groups engaged in the formation of the hydrogen bonds [62].

The peak detected at 1426 cm^−1^ in the FTIR spectrum of hemp corresponds to the bending vibrations of C–H bonds from the CH and CH_2_ groups of cellulose, hemicellulose and lignin. The peak recorded at 1457 cm^−1^ in the FTIR spectrum of PP and in the FTIR spectra of the composites that contain PP corresponds to the bending vibrations of C–H bonds from the CH_2_ and CH_3_ groups of propylene [63]. The peak located at 1315 cm^−1^ in the FTIR spectrum of the hemp, attributed to the rocking vibrations of the C–H bonds from the CH_2_ groups of cellulose, was not found in the FTIR spectra of the rigid composites that contain hemp [64]. The peak recorded at 1026 cm-1 in the FTIR spectrum of hemp, attributed to the stretching vibrations of the C–O bonds (C–O–C glycosidic linkages from cellulose and hemi-cellulose, C–OH phenolic groups from lignin and C–O–C ether linkages from lignin, respectively), decreased greatly in intensity as the amount of hemp in the composites decreased [65]. The decrease in the peak intensity once the PP/H ratio had increased may be due to the steric hindrance that occurs as the amount of PP in the composite increases. The band located around 1094 cm^−1^ in the FTIR spectra of the polyester fibres, bicomponent fibres and hemp containing composites is attributed to the stretching vibrations of the C–O bonds from the ester groups of polyester, as well as to the stretching vibration of the C–O bonds from the ether groups of cellulose, hemicellulose and lignin. The peak located around 898 cm^−1^ is characteristic of the glycosidic β-linkage from the cellulose and hemicellulose. The peak detected around 872 cm^−1^ is attributed to the stretching vibrations of the C–O bond from the polyester. The peak recorded around 840 cm^−1^ is assigned to the out-of-plane stretching vibrations of the C–H bonds from the aromatic rings present in the lignin [65]. The presence of the peaks located at 721, 723 and 725 cm^−1^ is related to the out-of-plane stretching vibrations of the C–H bonds from the aromatic ring [66].

### 3.5. SEM Analysis

**Figure 11:**
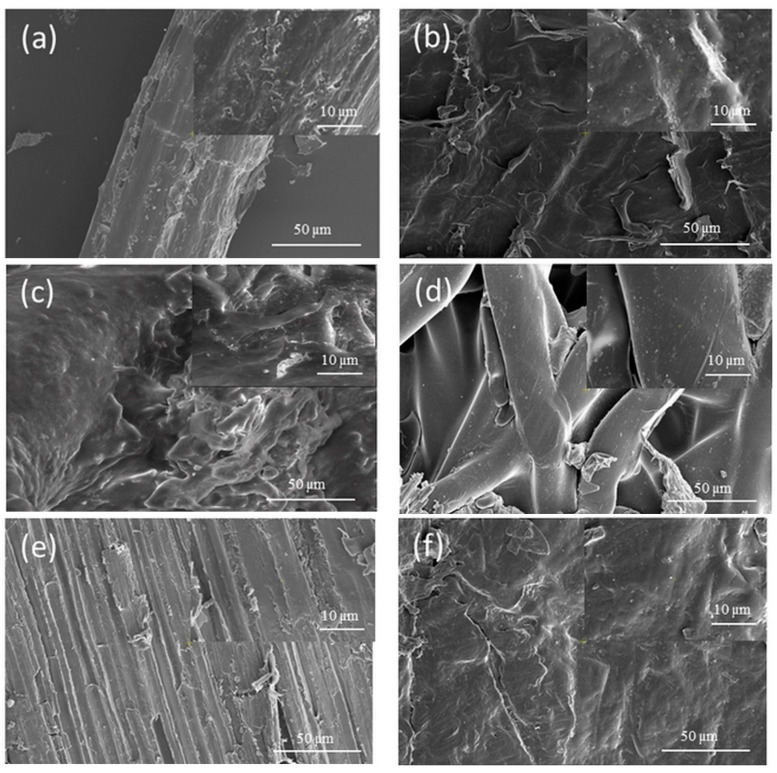

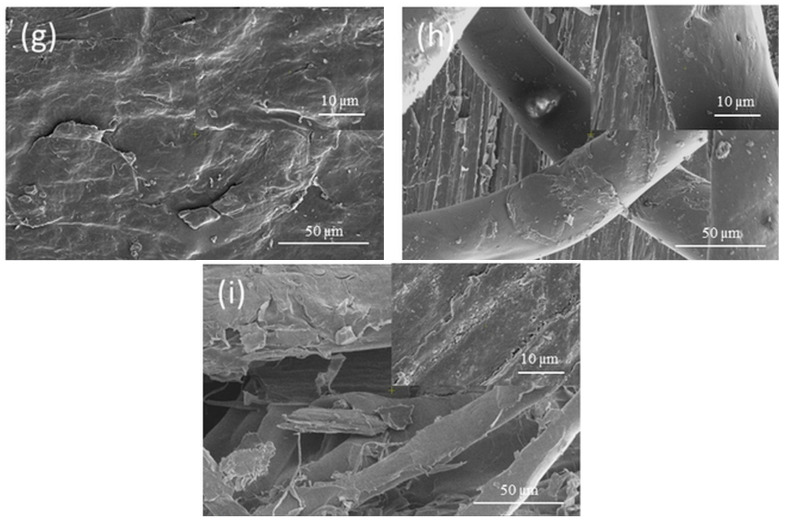
SEM images of (**a**) hemp fibres, (**b**) polypropylene, (**c**) polyester, (**d**) bicomponent fibres; (**e**) 75H25PP, (**f**) 50H50PP, (**g**) 25H75PP, (**h**) 50H50PES and (**i**) 50H50BI.

The SEM images of the hemp fibres, polymeric fibres and composite materials are presented in Figure 11.

The matrices of PP, PES and BI, the reinforcing agent, and the obtained composite materials were morphologically investigated. The images were recorded at 1000× and 5000×.

From Figure 11a it can be observed that the size of the hemp fibres is about 50 μm and their surface is rough. PP and PE matrices (Figure 11b,c) have a specific compact, homogeneous structure. In the case of the BI matrix, it can be observed that the fibres have a size of approximately 20–30 μm, and the surface of the fibres is smooth. In order to highlight the influence of the matrix, the morphology of the composite materials with 50% reinforcing agent (hemp) and 50% matrix (PP) was analysed (Figure 11f,h,i). From a morphological point of view, good adhesion between components implies a compact structure, in which the reinforcing agent is evenly distributed in the matrix. In Figure 11e–g, the influence of the hemp percentage on the morphology of the H–PP composite can be observed. At a rate of 25–50% hemp, it can be noticed that the fibres are well integrated into the PP matrix. When the percentage of hemp increases to 75%, some of the fibres are not embedded in the matrix. The results indicated by the SEM images are also confirmed by the physico-mechanical tensile strengths, which increased in the first stage for the composites that contain up to about 50% hemp, and after that they exhibited a decreasing tendency.

### 3.6. Chromatic Measurements

**Figure 12:**
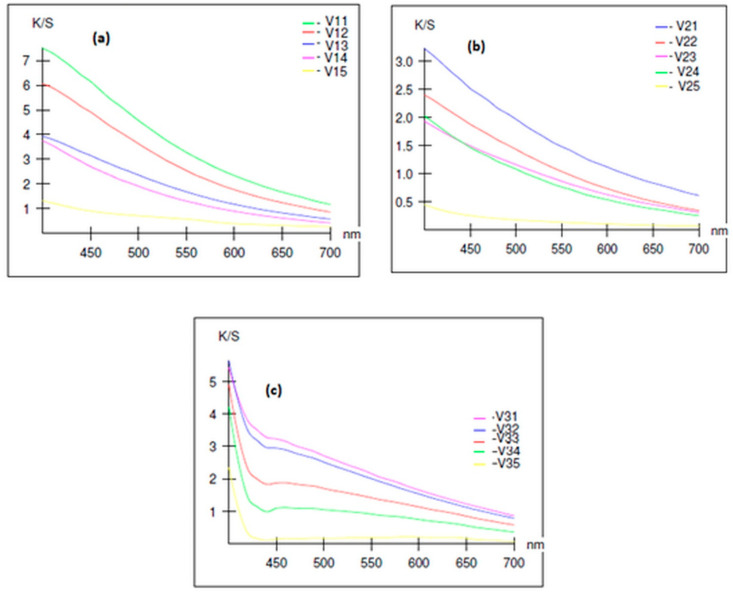
Variation in the colour strength of composite materials obtained from: (**a**) polypropylene/hemp fibres; (**b**) bicomponent/hemp fibres; (**c**) recycled polyester/hemp fibres.

Chromatic measurements of composite materials were performed on the entire visible spectrum and were evaluated by colour intensity (K/S). The obtained results are presented in graphic form in Figure 12. Following the processes of obtaining composite materials, the initial mixture underwent a series of chromatic changes. Under the action of temperature and pressure, the chemical fibre (PP, BI or PES) melted and diffused throughout the mass of hemp fibre.

By cooling, a homogeneous material is obtained whose chromatic characteristics will be influenced by the ratio between the hemp fibre and matrix. In all the analysed variants, the colour intensity of the samples increased with the increase in hemp fibre content, and decreased with the increase in the polymeric fibre content used as matrices.

## 4. Conclusions

In this paper, a comparative study was performed on composite materials obtained by heat pressing from hemp fibres and three polymeric matrices (polypropylene, bicomponents, recycled polyester). The influences of the matrix and of the polypropylene/ hemp ratio were investigated. By analysing the results obtained, the following can be concluded:

- the highest tensile strengths were obtained for composites containing 30% to 50% hemp fibres;
- the elongation at break, which is about 71.5% for the sample consisting of 100% hemp, falls below 10% for the composites that contain 25% polymer matrix;
- thermal stability decreases as the amount of hemp in the composite increases (for the same mass losses, the decomposition temperature decreases significantly for composites containing quantities higher than 50% hemp), and with regard to the influence of the matrix, for the same mass losses, the decomposition temperature of the composites decreases in the order of 50H50PES > 50H50BI > 50H50PP;
- the SEM images indicate the presence of gaps in the composites that contain a quantity of hemp less than 50%, which could explain the better adhesiveness between the two components of these composites;
- the colour strength of the composite materials increased with the increase in the amount of hemp fibres, and decreased with the increase in the amount of polymeric fibres used as matrix.

## Figures and Tables

**Table 1:** Characteristics of the fibres used to obtain composites.

Fibres	Characteristics			
	Title (dtex)	Tenacity (cN/tex)	Elongation (%)	Fibre Length (mm)
Hemp	102	39.5	1.8	50–250; 50/60
Polypropylene	6.7	27	100	60
Polyester	19	28.5	50.80	38/64
Bicomponent	6.7	28	52	51

**Table 2:** Working variants.

Variant	Blend	Variant	Blend	Variant	Blend
V 1.1	100H	V 2.1	100H	V 3.1	100H
V 1.2	75H25PP	V 2.2	75H25BI	V 3.2	75H25PES
V 1.3	50H50PP	V 2.3	50H50BI	V 3.3	50H50PES
V 1.4	25H75PP	V 2.4	25H75BI	V 3.4	25H75PES
V 1.5	100PP	V 2.5	100BI	V.3.5	100PES

**Table 3:** Thermogravimetric parameters of the investigated samples.

Sample	Stage	T_onse_ °C	T_peak_ °C	T_endset_ °C	Mass Loss %
100H	I	303	359	377	57.74
	II	377	446	499	11.31
100PES	I	399	435	461	89.33
100BI	I	396	436	462	84.98
	II	462	601	664	14.81
100PP	I	358	437	453	99.39
50H50BI	I	283	343	357	21.43
	II	357	432	454	50.38
	III	454	546	654	23.95
50H50PES	I	328	345	401	9.68
	II	401	435	458	79.97
25H75PP	I	261	280	321	7.46
	II	321	340	384	15.69
	III	384	440	463	74.10
50H50PP	I	266	342	362	43.20
	II	362	394	424	42.61
	III	424	465	535	14.55
75H25PP	I	261	339	361	44.59
	II	361	467	483	31.18

**Table 4:** Degradation temperature (°C) and mass loss (%) of hemp, polymers and composites.

Mass Loss %	100H	75H25PP	50H50PP	25H75PP	100PP	100BI	50H50BI	50H50PES	100PES
10	275	276	279	315	386	405	322	403	413
15	307	296	296	335	396	411	340	411	417
25	331	321	320	373	411	420	385	422	424
50	356	346	360	411	434	436	429	437	438

**Table 5:** DSC data for PP and its composites.

	First Heating		Cooling			Second Heating		
Sample	T_m_ (°C)	ΔH_m_ (J/g) *	Tc (°C)	ΔH_c_ (J/g)	Χc (%)	T_m_ (°C)	ΔH_m_ (J/g) **	Χc (%)
100H		–	–	–	–		–	
75H25PP	161.58	32.35	115.55	39.97	77,23	161.58	35.68	68.94
50H50PP	163.28	41.96	115.30	54.33	52.49	162.25	46.86	45,27
25H75PP	164.26	59.19	115.20	72.33	46,58	163.20	63.42	40,85
100PP	167.54	74.40	112.5	93.56	45,19	164.74	81.67	39.45

* First heating. ** Second heating.

**Table 6:** DSC data for hemp and its composites.

Sample	ΔH_m_ (J/g) *	ΔH_c_ (J/g)	ΔH_m_ (J/g) **	T_g_ (°C) ***
50H50BI	2.26	3.40	2.34	61.91
100BI	31.16	18.10	26.83	70.38
50H50PES	42.72	36.32	31.25	74.58
100PES	50.70	39.68	33.81	77.52

* First heating. ** Second heating. *** Glass transition temperature (midpoint).

## Data Availability

We have uploaded datasets analyzed during study on a shared drive. https://drive.google.com/drive/folders/1Rk-tse9mdti_Cr96kgfqdZGwtK5GqWyP?usp=sharing.
